# A Scalable Low-Input Redox Proteomics Workflow Enables High-Throughput Analysis of Cysteine Oxidation in 96-Well Cell Cultures

**DOI:** 10.1016/j.mcpro.2026.101641

**Published:** 2026-08-17

**Authors:** Adam M. Kabza, Rosey Chu, Jesse Trejo, Xiaolu Li, Matthew J. Gaffrey, David Hall, Wei-Jun Qian, Tong Zhang

**Affiliations:** Biological Sciences Division, Pacific Northwest National Laboratory, Richland, Washington, USA

**Keywords:** redox, cysteine oxidation, proteomics, enrichment, low-input, high-throughput

## Abstract

Reversible oxidation of cysteine residues (redox modifications) plays a crucial role in regulating protein function, signaling, and cellular homeostasis. These dynamic modifications act as molecular switches that transduce redox signals and modulate stress responses, metabolism, and pathogenesis. Redox proteomics enables systematic profiling of these modifications, quantifying the oxidation levels of tens of thousands of cysteine sites across the proteome and providing rich data to understand redox-regulated networks. However, conventional redox proteomic workflows are often limited by low-throughput and high sample requirements. Here, we present a high-throughput sample processing workflow for redox proteomics analysis from as little as 2 μg of protein. The workflow integrates 96-well plate–based cell culture, lysis, digestion, and cysteine-peptide enrichment, substantially increasing throughput and reducing hands-on processing time. Incorporating field asymmetric ion mobility spectrometry (FAIMS) further enhances redox proteome coverage by removing singly charged species in low-input samples, thereby increasing the signal of cysteine-containing peptides. Applying the workflow to RAW264.7 cells cultured in 96-well plates (40,000 cells per well), DIA identified >10,000 cysteine sites, and revealed a global increase in cysteine oxidation upon diamide treatment. To assess robustness, we repeated the 96-well experiment across three independent batches processed on different days and observed consistent coverage, reproducible quantification, and comparable diamide-induced oxidation of heat shock proteins, transcription factors, and protein kinases. Together, this workflow and new data acquisition scheme enable comprehensive redox proteomics from minimal inputs, paving the way for high-throughput sophisticated studies of redox modifications in cell signaling, disease, or large-scale screening of redox-modulating compounds.

## Highlights

• A highly sensitive SP3–RAC workflow for low-input redox proteomics.
• Plate-based lysis, digestion, SPE, and enrichment enable HT redox proteomics.
• Robust cross-batch reproducibility and consistent diamide-sensitive redox signatures.

## Graphical Abstract

**Figure:**
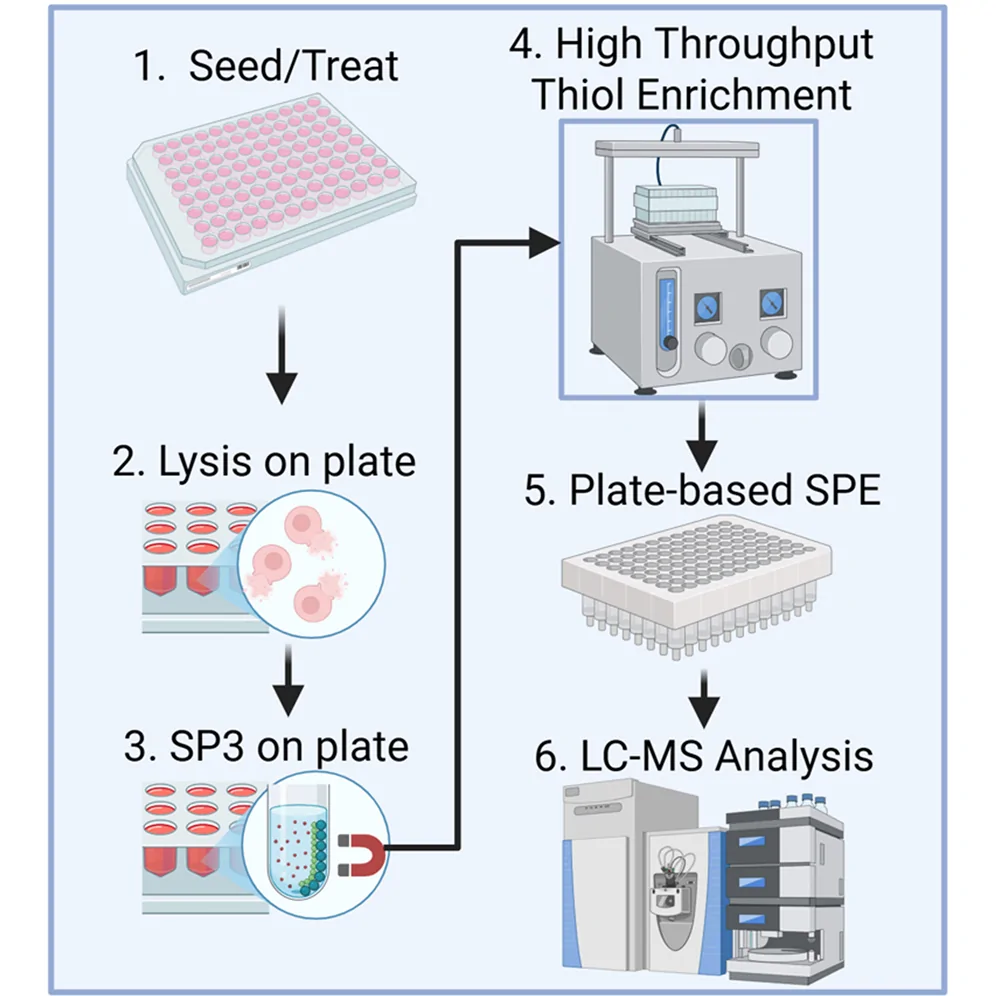

## In Brief

We developed a 96-well plate–based SP3–RAC workflow that enables high-throughput redox proteomics from a few μg of protein, allowing redox-state profiling directly from cells cultured in 96-well plates. Coupled to FAIMS and DIA, the method increases sensitivity in low-input samples and quantifies >10,000 cysteine sites in RAW264.7 cells. Across three independent plates processed on different days, the workflow produced reproducible coverage and consistent diamide-induced oxidation signatures, enabling scalable studies and screening of redox-modulating perturbations.

Maintaining reduction-oxidation homeostasis is critical for normal cell functions (1, 2, 3, 4), and alterations in the redox balance of cells is a common response to stimuli including chemical insult and disease. Decades of research in redox biology has established a central role of reversible modifications on protein cysteine thiols in redox signaling (3, 4, 5, 6, 7, 8). Cysteine accounts for 2.3% of all amino acids in proteins and often serves as the active or regulatory sites in enzymes. The cysteine thiol (-SH) group is highly reactive and can undergo a variety of reversible post-translational modifications (PTMs). Such redox PTMs act as molecular switches to regulate protein structure, activity, localization, and interactions, enabling rapid and reversible control of signaling pathways under both physiological conditions and in response to changes in cellular redox environment (8, 9, 10).

With the advances of mass spectrometry (MS)-based proteomics, the oxidation state of cysteines can be quantified at the proteome scale in biological systems (11, 12, 13, 14). One main challenge in redox proteomics is to detect the often low-abundance peptides harboring oxidized cysteine residues, due to the low occurrence of cysteines in the proteome and an overall reducing environment in the cell. Consequently, most modern redox proteomics approaches including OxICAT (15), Oximouse (16), CysTMT (17, 18), IodoTMT (19), and thiol-affinity resin-assisted capture (RAC) (11, 12) employ enrichment steps to improve sensitivity and coverage of the redox proteome. OxICAT utilizes two isotopically labeled iodoacetamide species, which also include a biotin handle for downstream enrichment *via* streptavidin beads. Similarly, CysTMT attaches a tandem mass tag (TMT) as well as biotin to cysteine residues. Oximouse uses cysteine-reactive phosphate tags (CPTs) with a phosphorylated epitope for enrichment *via* immobilized metal affinity chromatography. RAC thiol enrichment stands out as it employs a thiol exchange reaction to covalently bind cysteine-containing peptides to the resin during the enrichment step. Moreover, TMT labeling can be incorporated into the RAC protocol seamlessly, making this one of the most powerful approaches for redox proteomics investigations of cysteine PTMs.

Despite these advances, there are two major roadblocks in redox proteomics sample preparation: high sample input requirement and low sample throughput. This is largely due to the labor-intensive enrichment step that typically require large amounts of input. For instance, 200 μg of crude proteins per sample are used in our standard RAC redox proteomics workflow, and it takes ∼6 h of intensive hands-on work to enrich 12 samples (12). Using the isoTMT approach, Pimkova *et al*. identified ∼4000 cysteine-containing peptides from 400,000 sorted hematopoietic stem and progenitor cells (∼20 μg of protein) (20). In the PACREDOX approach, endogenous reduced and oxidized thiols are differentially labeled with iodoacetamide and methylmethanethiosulfonate, respectively, without enrichment; applying this method to 90 μg of cardiac tissue protein enabled identification of ∼4000 cysteine-containing peptides by data-independent acquisition (21). Lilla (22), Martinez-Val (21), and Xiao (23) *et*. *al*. perform “low-input” by leveraging TMT labeling to achieve a higher pooled peptide inputs (>30 μg) for the enrichment steps, requiring up-front input of 10 to 90 μg peptides from individual samples prior to the pooling. Desai *et al* utilizes SP3 to make their method high throughput but continues to require very large sample inputs of 400 μg for enrichment (24). This mass requirement together with the limited throughput (<40 samples per week) greatly impedes its application to large-scale and clinical studies.

Our aim in this work is to address these challenges by developing a low-input, high-throughput redox proteomics capability. To perform redox proteomics with less than 10 μg of proteins, we evaluated key parameters in RAC enrichment, including peptide input and resin amount, as well as the data acquisition schemes during liquid chromatography (LC)-MS. We also developed a new 96-well plate-based RAC enrichment for high-throughput redox proteomics. This novel approach enables proteomic profiling of redox PTMs from cell cultures on 96-well plates with as low as 10,000 cell density seeding. It also improves sample throughput, from 12 samples processed manually over the course of 3 days, to 96 samples (scalable to multiple plates with hundreds of samples) processed in 2 days with a >50% reduction in hands-on processing time. The robustness of the workflow was validated in RAW264.7 cells seeded at 40,000 cells per well, in which three independent experimental batches produced comparable redox proteome coverage and consistent diamide-induced shifts in cysteine oxidation. These advancements enable the expansion of redox proteomics workflows to large-scale and sample-limited studies allowing library screening of redox-modulating compounds for their therapeutic potentials.

## Experimental Procedures

### Cell Culture and Treatment

For initial testing and development experiments, we utilized β-TC-6 cells cultured in RPMI-1640 with 11.1 mM glucose (Gibco), 10% (vol/vol) fetal bovine serum (FBS), 1% GlutaMax (vol/vol) and 1% anti-micotic/anti-mytotic solution (25). Cells were plated at ∼80% confluency the day before treatment. Cells were treated by removing media and replacing it with 8 ml of fresh media containing - 100 U/ml interleukin-1 beta and 250 U/ml interferon gamma (both by Peprotech). After 4 h of treatment, the media was removed, and the cells were washed twice with ice-cold PBS with or without 100 mM N-ethylmaleimide (NEM) for total oxidation or total thiol content, respectively. Cells were either immediately lysed or frozen at −80 °C until further analysis. Protein extraction, single-pot solid-phase enhanced sample preparation (SP3)-based cleanup and digestion, and peptide desalting were described before (25).

For cellular experiments we utilized Murine RAW 264.7 macrophages cultured with RPMI-1640 media. The complete medium was prepared by the addition of 10% (v/v) fetal bovine serum (FBS), 1% GlutaMax (v/v) and 1% anti-mycotic/anti-mitotic solution. All cell cultures were maintained in 10 cm dishes at 37 °C under 5% CO_2_ and passaging was conducted at either 1:3 or 1:6 dilutions.

For 96-well culturing for low-input experiments, cells were detached from the 10 cm dish by gentle scraping, counted using a cell counter, and seeded at either 10,000 or 40,000 cells/well 24 h before treatment and subsequent lysis. After 24 h, cells are treated with either 1 mM diamide or control (PBS) at 37 °C for 30 min (26) followed by two brief washes of PBS containing 125 mM NEM to block reduced thiol groups. Samples were either immediately lysed with buffer containing NEM or frozen at −80 °C until further analysis.

### Sample Preparation for Redox Proteomics

Proteomics sample preparation from cell culture was performed using a modified SP3 protocol. The SP3 beads were prepared as follows. First, both hydrophobic and hydrophilic SP3 beads (Cytiva Lifesciences, cat. 45152105050250 and 65152105050251) were equilibrated to 25 °C (∼1 h), resuspended, and mixed at a 1:1 ratio (100 μl of each in a 2 ml Eppendorf tube). The beads were placed on a magnetic stand for 2 min and the supernatant was removed. Beads were then washed sequentially with 2 ml of 50 mM HEPES (pH 9.1), 200 mM NaCl, 1 ml 1 M NaCl, and ultrapure water, and were suspended in 200 μl of ultrapure water at a concentration of 50 μg/μl.

For cells cultured in 96-well plates, 50 μl of lysis buffer (250 mM MES, pH 6, 5% SDS, 1% Triton-X-100, 125 mM NEM, 10 mM EDTA, prepared fresh) was added to each well and pipetted up and down repeatedly to achieve a homogenous lytic suspension. Protein concentration was determined by taking 10 μl of the lysate for bichinoic acid (BCA) assay (ThermoFischer Scientific). Protein lysates were then subjected to cleanup using SP3. To each cell lysate, beads at a 30:1 ratio (mass ratio of bead: protein) were added, and the reaction volume was normalized to 50 μl with lysis buffer. After incubation for 10 min at 25 °C with shaking at 1000 rpm, 50 μl of absolute ethanol was added. Samples were then placed on a magnetic stand for 5 min, and the supernatant was removed. The beads were washed four times with 100 μl of 80% ethanol, and each wash was incubated for 5 min with shaking at 1000 rpm. On-bead digestion was performed by adding 100 μl of digestion mix containing trypsin and lysC (enzyme-to-protein ratio of 1:100 (w/w) for both) and incubating at 37 °C for 18 h with shaking at 1000 rpm. After collecting the peptides in the supernatant, the beads were washed with 100 μl of 250 mM HEPES (pH 7.7) containing 5% acetonitrile (ACN), and the supernatant was combined with the first elusion. The combined eluates were placed on a magnetic rack to remove any residual beads. The supernatant was dried in a SpeedVac, followed by solid-phase extraction (SPE) using an OASIS Max 96-well μElution plate.

For SPE, samples were processed using an Oasis MAX 96-well μElution plate according to the manufacturer’s instructions. The plate was conditioned with two 200 μl washes of 100% methanol and equilibrated with two 200 μl aliquots of ultrapure water. Samples were loaded onto the plate and passed through the sorbent matrix by centrifugation, followed by four washes with 200 μl of 5% ACN (v/v) in water containing 0.1% TFA. Peptides were eluted sequentially with 100 μl of 80% ACN in water with 0.1% TFA and 100 μl of 100% ACN containing 0.1% TFA into a 1 ml 96-well collection plate. After SPE cleanup, samples were ready for thiol-affinity enrichment.

### Preparation of Thiol Affinity Resin

The synthesis of thiol affinity resin was performed as follows (27). First, 5 ml of Fast Flow 4 NHS-Activated Sepharose (Cytiva, cat. 17090601) was placed in a 10 ml spin column and centrifuged at 100 *g* for 2 min to remove isopropanol. Next, the resin was washed with 5 aliquots of 5 ml 1 mM hydrochloric acid, centrifuging between each wash. Then the resin was washed once with 5 ml of coupling buffer (0.2 M NaHCO_3_, 0.5 M NaCl, pH 8.3). During this wash, 175 mg of 2-(pyridyldithio)ethylamine hydrochloride was dissolved in 3.5 ml of the coupling buffer. Next, 3 ml of this solution was added to the resin and incubated for 2 h at 25 °C with shaking at 1000 rpm on a thermomixer. After reacting, the reaction buffer was removed by centrifugation and the resin was washed five times with 5 ml of coupling buffer. The unreacted NHS groups on the resin were quenched with 3 ml of 1 M Tris (pH 7.5) for 1 h at 25 °C with shaking at 1000 rpm. Next, the resin was washed with 5 aliquots of 5 mL ultrapure water followed by three washes of 3 mL isopropanol. Finally, the resin was suspended in 7.5 ml of isopropanol at a concentration of 200 mg/ml and stored at 4 °C for up to 9 months.

### Enrichment of Cysteine-Containing Peptides with Vacuum Manifold

Enrichment of cysteine-containing peptides using a vacuum manifold was performed as described (11, 27). Peptides were aliquoted into Eppendorf tubes in amounts ranging from 1 to 200 μg, dried in a SpeedVac, resuspended and reduced with DTT (concentration varies among experiments, with the optimized concentration of 5 mM) for 60 min on a Thermomixer. During this time, the thiol-activated 4 NHS-activated Sepharose resin was prepared. Per sample, 10 mg of resin was used if not stated otherwise. Peptides were loaded to the affinity resin, incubated for 120 min, washed, and eluted. The enriched peptides were dried on a SpeedVac (∼3–5 h), desalted by SPE using a C18 Omix 100 μl zip tip, and then eluted with 80% ACN and 0.1% TFA into an n-Dodecyl β-D-maltoside (DDM)-coated LC-MS vial. The vials were prepared by adding 12 μl of 0.01% DDM (w/v) into Waters LC-MS vials and speed-vac to dryness. Finally, samples were dried on a SpeedVac (∼2 h) and stored at −80 °C until LC-MS analysis.

### High-Throughput Well-Plate Enrichment of Cysteine-Containing Peptides

The high-throughput enrichment protocol was adapted from the standard enrichment workflow with several modifications to enable parallel processing in a 96-well plate. First, 10 mg of resin and reduced peptides were loaded to an AcroPrep 96-well plate with a polypropylene/polyethylene filter membrane (Cytiva, cat. 8127). To enable lossless sample incubation with the resin, the plate was sealed using a custom silicone mat molded from liquid silicone (Mold-Star 16 Fast, Smooth-On), cast with a 96-well plate as the template. Briefly, the custom silicone mat was prepared by pouring Mold-Star 16 fast liquid silicone into a tray to a thin layer of 5 to 6 mm and gently placing an AcroPrep 96-well plate onto the layer of silicone to form well-specific plugs. The silicone was allowed to sit for 2 h until the material was solidified, after which the mat was removed and excess silicone was cut with a knife. This sealing mat provided a tight and uniform seal across all wells, allowing secure incubation and complete recovery of samples at each step. After incubation, solvent washing steps were performed using a Presston positive pressure manifold (Agilent Technologies). For elution, the well plate was placed atop a collection plate (Thermo Fisher Scientific, cat. no. 260252), and positive pressure was applied to drive solvents through the filter membrane into a collection plate.

For plate-based SPE desalting, the procedure described above for the Oasis MAX 96-well μElution plate was used. The final eluates were transferred to DDM-coated LC-MS vials, and each well was rinsed with an additional 20 μl of 80% ACN with 0.1% TFA to minimize sample loss. Combined eluates were dried in a SpeedVac and stored at −80 °C until LC-MS analysis.

### Liquid Chromatography–Mass Spectrometry

Mass spectra for peptides were acquired on an Orbitrap Exploris 480 Mass Spectrometer (MS) (ThermoFisher Scientific) coupled to a Vanquish Neo ultra-high pressure liquid chromatograph (UHPLC) (Thermo Scientific). Peptides were first loaded onto a μPAC Neo Low-Load Trapping Columns (Thermo Fisher Scientific) and then separated on an in-house packed reversed-phase column at 45 °C (1.7-μm Waters BEH C18; 30 cm × 360 μm o.d. × 75 μm i.d with a fully integrated tip). Mobile phases consisted of 0.1% formic acid in water (A) and 0.1% formic acid in ACN (B), operated at a flow rate of 300 nl/min. Peptides were eluted with a gradient profile as follows (min:%B): 0:1.0, 2:1.0, 12:8.0, 117:25, 129:33, with a 13 min start delay for MS data collection. For experiments with a shorter 30 min gradient, the elution profile was (min:%B): 0:1.0, 2:1.0, 8:8.0, 30:25.0, 32:33.0, followed by a column and trap wash and equilibration, with a 13 min start delay for MS collection. For FAIMS experiments, all LC-MS settings were kept identical, except the FAIMS device was installed between the electrospray source and the mass spectrometer. The FAIMS was set to Standard Resolution mode, where the inner and outer electrodes are set to 100 °C and the FAIMS carrier gas is ultra-high purity N_2_ with a flow rate optimized to 3.8 L/min. In Standard Resolution mode, the asymmetric waveform values are DV = −5000 V and the entrance plate voltage = 250 V. Two compensation voltages of −40 V and −55 V cycled at 1 s each were used.

For data-dependent acquisition (DDA), the heated capillary temperature and spray voltage were 300 °C and 2.2 kV, respectively. Orbitrap MS1 spectra were collected from 300 to 1800 m/z at a resolution of 60,000, profile mode, and automated gain control (AGC) of 250%. Top 20 MS spectra were selected with an isolation width of 0.7 m/z and subjected to higher-energy collisional dissociation (HCD) at 30% energy, and the resulting the MS2 spectra were collected at a 30,000 resolution and centroid mode. A dynamic exclusion duration of 30 s was also set.

For data-independent acquisition (DIA) MS runs, Orbitrap MS1 spectra were collected from 380 to 980 m/z at a resolution of 120,000, profile mode, and AGC of 250%. Precursors were selected sequentially with an isolation window of 10 m/z and subjected to HCD collision with energy at 30%. The resulting MS2 spectra were collected at a 30,000 resolution using the centroid mode.

### Cross-Batch Performance Evaluation of the Plate-Based Low-Input Redox Proteomics

To assess method reproducibility and robustness, a biological triplicate experiment was performed using three independent 96-well plates prepared and treated on three separate days. For each of the three batches, 40,000 cells per well were seeded and treated as described (in the Cell Culture and Treatment section). After plate-based SP3 sample processing, reduction, and enrichment of cysteine-containing peptides, 6 replicates per experimental condition (control and diamide treatment) were analyzed by DIA with the optimized FAIMIS conditions.

### Data Analysis, Statistics, and Bioinformatics

For DDA data, raw MS data were searched using MS-GF+ against a *Mus musculus* UniProt database (downloaded in March 2023 with 21,949 entries) for peptide identification (28). For missed cleavages allowed, the default value of −1 (no limit) was used. The mass tolerance of precursor ions was set at 20 ppm. Because MS-GF+ uses instrument/fragmentation-specific probabilistic scoring rather than a fixed fragment-ion mass tolerance window, a fragment-ion tolerance parameter was not provided during the search. For total thiol samples, no cysteine modifications were specified, while methionine oxidation was considered a variable modification. For total oxidation samples, NEM alkylation of cysteine residues was set as a dynamic modification in addition to methionine oxidation. MS-GF+ processed results were subjected to filtering using PepQ and MSGFDB SpecE value thresholds of 0.01 and 1E-8, respectively. Next, peptide spectrum matches (PSMs) were aggregated to the peptide level, and a false discovery rate (FDR) <1% at the unique peptide level was applied. Enrichment selectivity was defined as the percentage of cysteine-containing peptides over all unique peptide identifications.

For the initial DIA dataset on the diamide-treated cell culture samples, raw MS spectra were searched using DIA-NN (version 2.3.1) (29). The same *M*. *musculus* protein database used for the DDA search was employed. Variable modifications included methionine oxidation, N-terminal acetylation, and NEM alkylation of cysteine. Trypsin was specified as the digestion enzyme and two missed cleavages were allowed at maximum. The mass accuracy for both precursor and fragment ions were set to automatic in DIA-NN; DIA-NN calibrated mass errors from the data and used the optimized values for searching. An FDR threshold of 1% was used for both the peptide and the protein group levels. To assess modification-specific error rates for CysNEM identifications, DIA-NN was run with decoy reporting enabled (--report-decoys), and per-run PTM-specific FDR for CysNEM peptides was calculated from the resulting report.parquet output by comparing target and decoy CysNEM peptide identifications at the applied q-value threshold. For the three-plate DIA dataset that quantitatively evaluates batch-to-batch performance of the workflow, raw MS spectra were search using Spectronaut (30) (Biognosys, V20) because datasets with multiple CVs do not need to be deconvoluted in Spectronaut. DirectDIA was used, with the same database and modifications as described above. Default mass accuracy tolerance settings were used (Dynamic for both calibration search and main search; 1 for MS1 and MS2 correction factor). The FDR was set to 1% at both peptide and protein levels.

For statistical analyses, the raw intensity of peptide output was log_2_-transformed, and data of cysteine-containing peptides was normalized by the median values of the non-cysteine containing peptides in the same sample. Data was then rolled up to the cysteine site using an in-house R script. Student’s *t* test was used to determine the statistical significance of cysteine sites between the diamide-treated and the control groups, requiring at least two valid quantification values per group. Proteins of peptides showing significant differences were subjected to enrichment analysis using the R package clusterProfiler (31). Protein-protein interaction analysis was performed using the R package STRINGdb (32). Known interactions were retrieved from STRING (v11.5) and filtered to retain high-confidence edges (combined score ≥700). The filtered interaction set was converted into an undirected tidygraph network, and node degree (number of connections) was used to identify the top 30 hub proteins. The network was visualized in graph using a force-directed layout. For transcription factor and protein kinase analyses, the curated databases were downloaded from the AnimalTFDB (33) and the Epithelial Systems Biology Laboratory. To evaluate whether detection of cysteine-containing peptides is associated with protein expression level, the aggregated peptide quantification results from Spectronaut were split into cysteine-containing and non-cysteine peptides, representing the cysteine-oxidation readout and total protein abundance, respectively. For each subset, intensity values across all samples were summed per protein group. Next, a per-protein mean across runs was calculated and used to rank the proteins. The cysteine-oxidation rank and mean intensity were subsequently compared with the corresponding protein-abundance rank and mean intensity using scatter plots and Pearson correlation.

### Experimental Design and Statistical Rationale

**Fig. 1:**
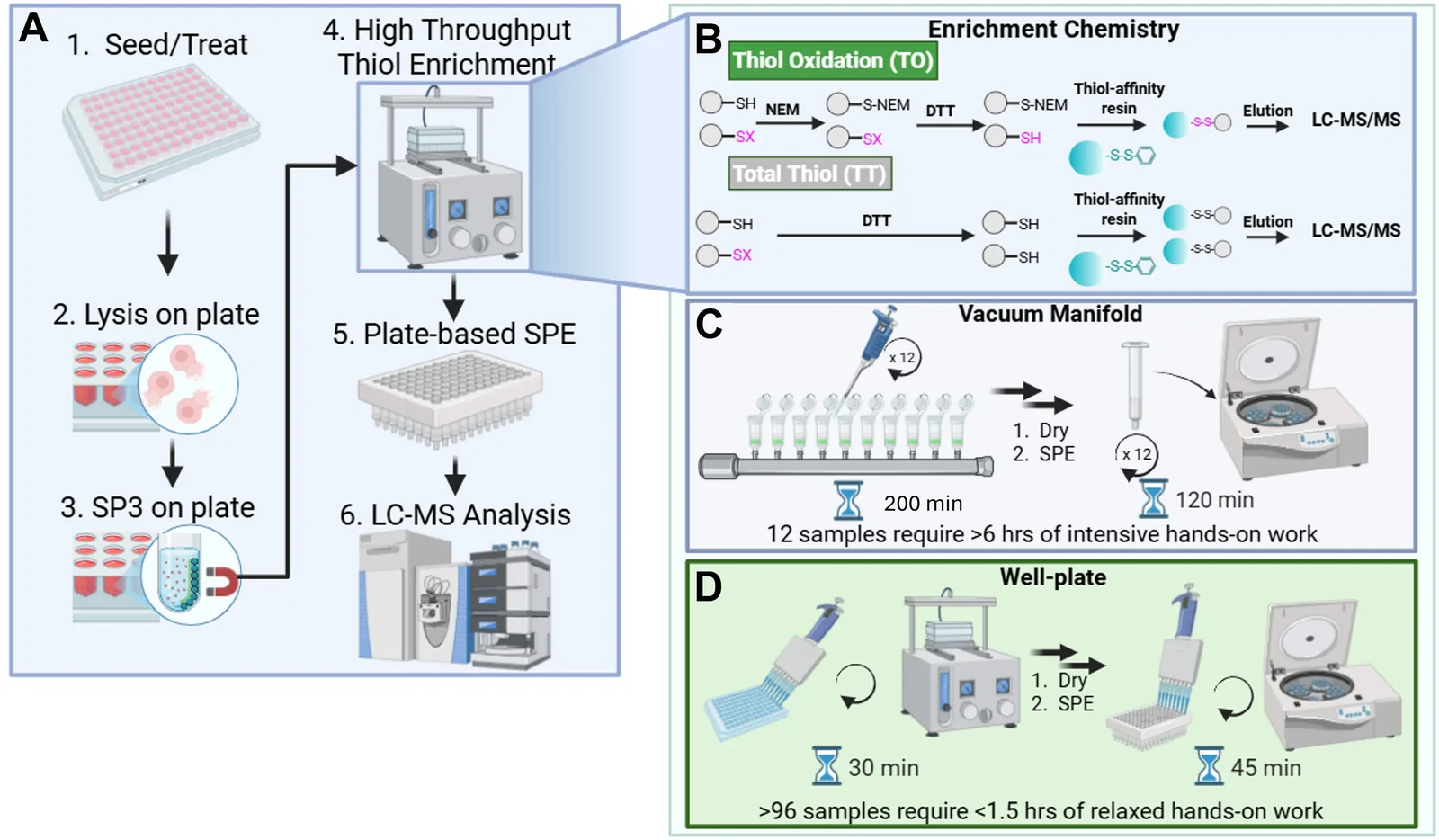
**High-throughput redox proteomics sample preparation.***A*, flow chart of the high-throughput redox proteomics workflow. *B*, chemistry of thiol-containing peptide enrichment *via* thiol–disulfide exchange reaction. *C*, the manual enrichment protocol using a vacuum manifold. Samples are processed in individual tubes/columns, which limits throughput to ∼12 samples per day and requires extensive repetitive pipetting. *D*, the well-plate method for high-throughput semi-automated enrichment. This protocol achieves a 10-fold increase in throughput and reduces hands-on work by around 3-fold.

**Fig. 2:**
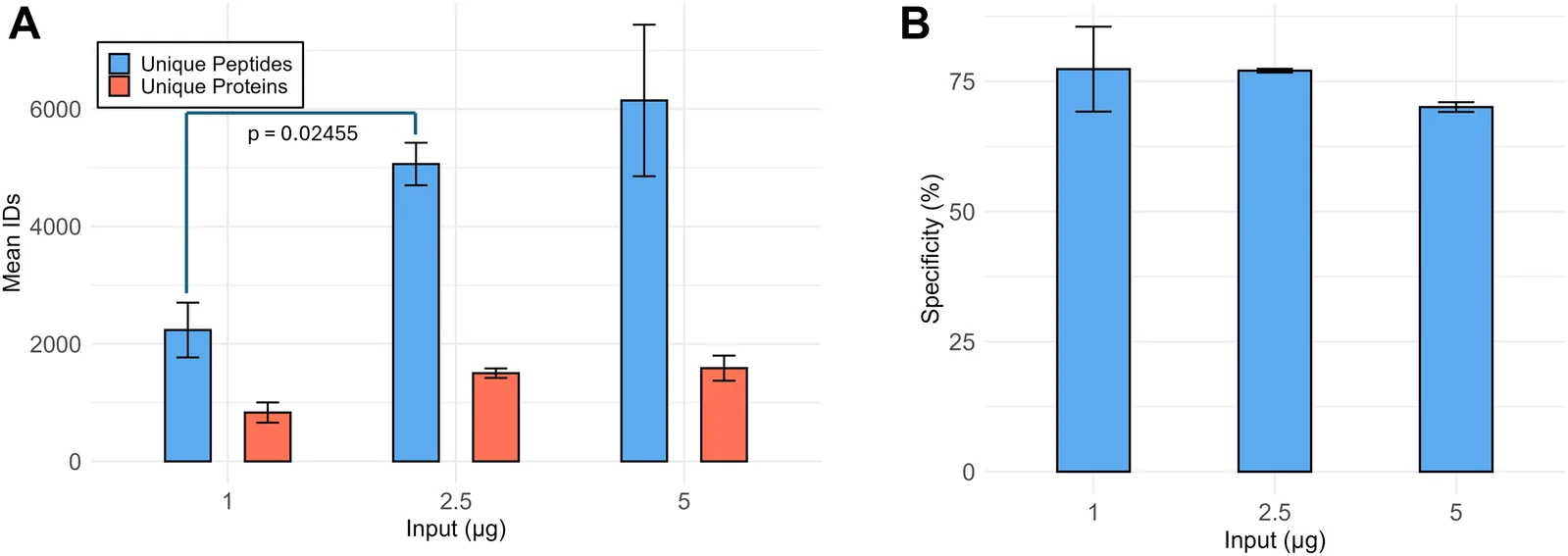
**Performance of low-input redox proteomics.***A*, cysteine-containing peptide and protein identifications from low-peptide input samples. Data are presented as mean ± SD. Statistically significant differences between the input groups (*p* < 0.05) are indicated. *B*, enrichment selectivity (number of cysteine-containing peptides relative to all peptide identifications) at lower input amounts.

**Fig. 3:**
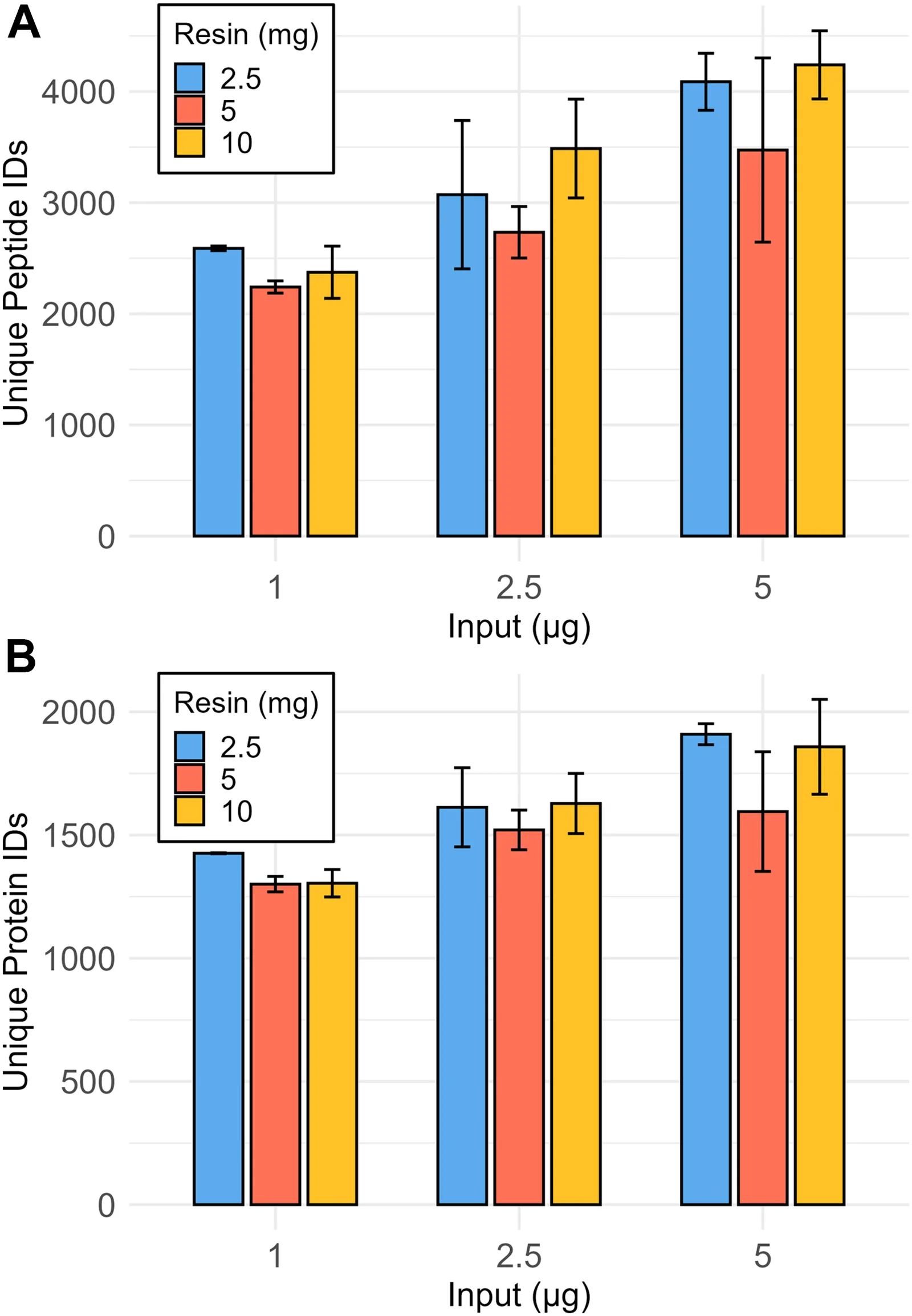
**Optimization of enrichment resin amount.***A*, cysteine-containing peptide identifications from low-peptide input samples at varying resin amounts. *B*, the number of corresponding proteins from the unique cysteine-containing peptides from the same dataset. Data are presented as mean ± SD.

**Fig. 4:**
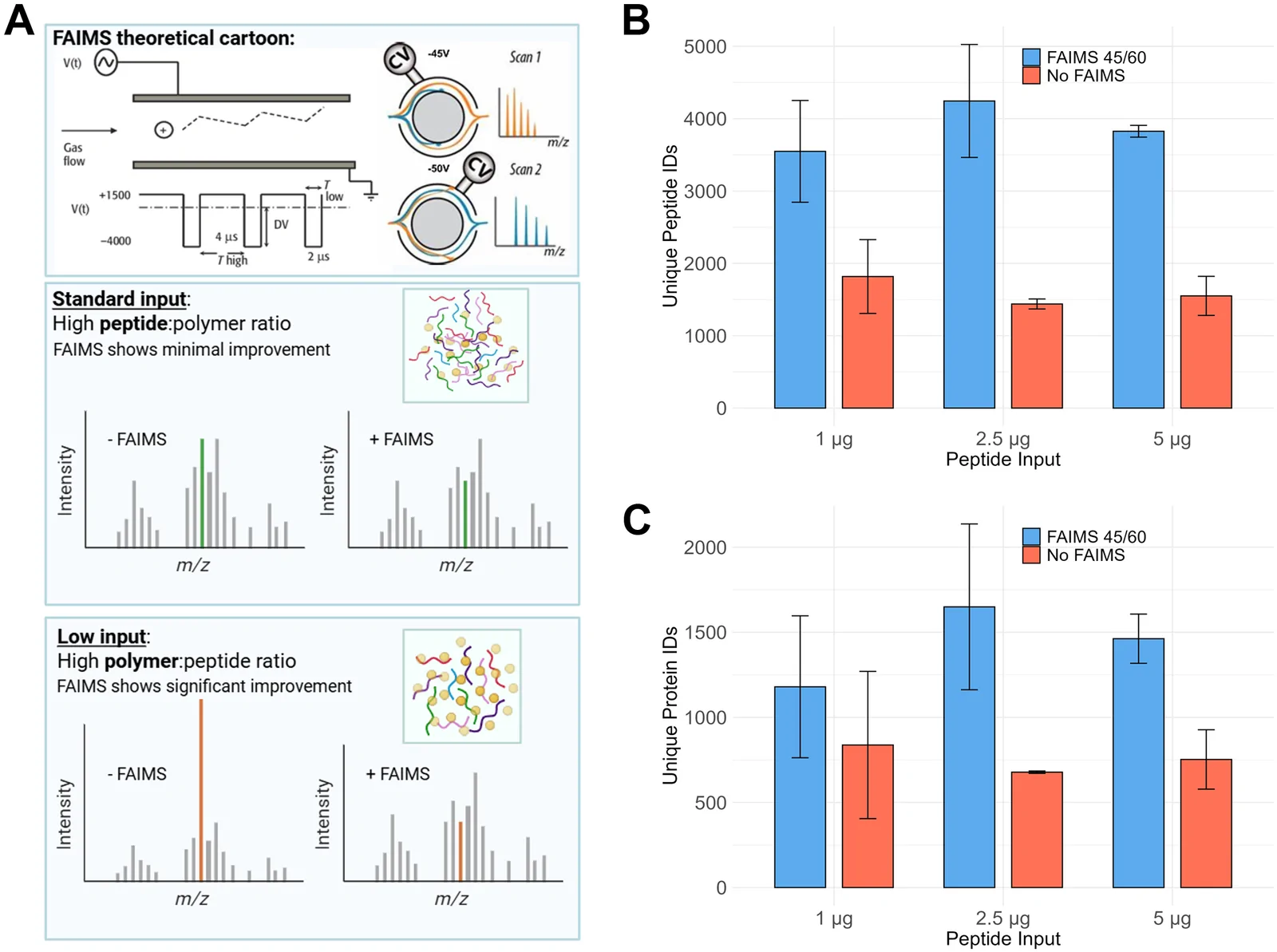
**FAIMS improves identification depth in low input samples.***A*, FAIMS uses compensation voltage (CV) for gas-phase separation and is effective in removing singly charged species (*top panel*). While singly charged ions are crashed into the central cylinder, peptides with higher charges pass the electric field into the MS. Theoretical rationales of the FAIMS effect on standard input (*middle panel*) and low input samples (*bottom panel*) for redox proteomics are shown. *B*, the effect of FAIMS with two-step CVs on cysteine-containing peptide identification at various sample input amounts. *C*, the number of corresponding proteins from the unique cysteine-containing peptides from the same dataset as in (*B*). In (*B*) and (*C*), data are presented as mean ± SD.

Initial workflow development and optimization experiments were performed using murine β-TC-6 cell lysate (Fig. 1, Supplemental Fig S1), as in our previous PTM proteomics method development work (25). Technical replicates from the same lysate were conducted to assess the impact of factors such as input amount, DTT concentration, resin amount, and FAIMS conditions on the performance of the workflow. A Student’s *t* test was used to compare the groups, and results were considered statistically significant when *p* < 0.05 and labeled in the figures (Fig. 2, Fig. 3, Fig. 4, Supplemental Figs S2 and S3). RAW 264.7 cells treated with diamide were used for low-input, plate-based workflow evaluation because of the well-studied biological effects of diamide in RAW 264.7 cells. An initial set with only one plate was used to test data acquisition schemes (DDA *versus* DIA). Finally, reproducibility was assessed using three independent 96-well plates prepared on three separate days (three batches). For each batch, 6 biological replicates per condition were analyzed by LC–MS. This design incorporated multiple independent batches (plates/days), allowing the assessment of between-batch reproducibility. In addition, multiple wells per condition increased precision for estimating variance and supported site-level statistical testing.

## Results and Discussion

### Developing a Low-Input and High-Throughput Redox Proteomics Workflow

To address two major limitations of traditional redox proteomics, namely high sample input requirements and low experimental throughput, we developed a 96-well plate–based workflow that integrates cell culture, sample preparation, and redox enrichment into a single scalable platform (Fig. 1*A*). The plate-based cell culture enables parallel generation of multiple experimental conditions with low input requirements per well (*e.g.*, <50,000 cells). Samples are processed using an automated SP3 workflow, which was compatible with low-input sample processing (34) and effective for redox proteomics using standard input amounts (*e.g*., 200 μg) (25). Coupling this with 96-well plate–based redox enrichment markedly increases sample throughput, enabling simultaneous processing of up to 96 samples. Because protein thiols can be oxidized into various reversible forms, we applied a strong generic reducing agent, DTT, to reduce all reversibly oxidized thiols into free thiols prior to enrichment and used total oxidation of protein thiols in this development (Fig. 1*B*). Key parameters of the RAC enrichment, including peptide input amount, DTT concentration during reduction, and resin amount, were iteratively screened (detailed below). Compared with our previous manual vacuum manifold–based enrichment, which required >6 h of hands-on time for 12 samples (Fig. 1*C*), the new plate-based system enables enrichment of 96 samples with less than 1.5 h of hands-on effort (Fig. 1*D*).

A notable innovation of the workflow is the use of 96-well plate for enrichment of cysteine-containing peptides. Compared to the traditional vacuum manifold-based enrichment, this method introduced several innovations to improve parallel processing and ensure reproducibility across individual wells. First, instead of individual spin columns, an AcroPrep 96-well filter plate was used as the reaction vehicle, enabling simultaneous handling of up to 96 samples. Second, the tedious washing steps were performed using a positive pressure manifold rather than vacuum, which provided more consistent flow rates and better scalability. Because the filter plates lacked a commercially available sealing during incubation, we fabricated a custom silicone sealing mat molded to the dimensions of the plate (Supplemental Fig. S1). This seal permitted lossless sample incubation and maintained uniform reaction conditions across all wells. Finally, the SPE cleanup step was also adapted for high-throughput operation by replacing individual zip-tip desalting with an Oasis Max 96-well μElution plate, allowing rapid and parallel cleanup of all enriched samples. Together, these design changes streamlined the enrichment workflow, significantly reduced hands-on time, and improved consistency for high-throughput redox proteomics.

### Workflow Optimization

For large-scale redox proteomics, we reason that the front-end 96-well plate format provides consistent culture environments and is compatible with automation for high-throughput scalable processing of hundreds of samples. Depending on cell type and size, a confluent well contains ∼25,000 to 60,000 cells, yielding ∼2 to 10 μg of protein per well. After SP3 digestion and SPE cleanup, <5 μg of peptide is expected for enrichment. To determine whether such low input amount is feasible for redox proteomics, we first performed enrichment with 1 to 5 μg of peptides using our standard protocol (11, 27), where lysis conditions, NEM blocking concentration, cleaning and enrichment steps were optimized for 200 to 500 μg protein inputs. Notably, 100 mM NEM was used for near-complete blocking of endogenous free thiols (11, 35), which is estimated to be ∼10,000X excess to proteins. Consequently, we did not change NEM concentration since it is in even higher excess at low-input conditions. Using DDA, we identified an average of 2236, 5062, and 6145 unique cysteine-containing peptides from the 1, 2.5 and 5 μg groups, respectively (Fig. 2*A* and Supplemental Table S1). These peptides can be mapped to ∼1000 (1 μg input) to 2000 unique proteins (5 μg input). An average of 75% enrichment selectivity was observed for all three input groups (Fig. 2*B*), suggesting the binding of non-cysteine peptides to the resin in low-input samples. Previous work in our group (25) demonstrates that with input amounts of 200 μg we are able to identify up to ∼10,000 unique peptides and ∼3000 unique proteins from cell culture sources. Thus, it is encouraging that reducing the input 40-fold to 5 μg still retains over 6000 unique peptides and ∼2000 unique proteins.

We next screened the DTT concentration in the reduction step. DTT was chosen because of its strong reducing power and its compatibility with proteomics workflow (*i.e*., easy to remove by C18 desalting). With DTT, our previous work had demonstrated a median of 10 to 15% oxidation (36, 37), consistent with a reducing cellular environment and estimations from other groups (16, 38). Given the significant reduction in sample input, we hypothesized that reducing the DTT concentration would improve binding, as the final DTT:peptide ratio during the binding reaction is much higher in low-input samples compared to standard input. We thus reduced 5 μg of peptides using DTT at 0.5, 1, and 5 mM and performed enrichment using the standard manual vacuum manifold. Interestingly, we observed that decreasing the DTT concentration did not improve peptide or protein identification (Supplemental Fig. S2, *A* and *B* and Supplemental Table S2). This suggests even a low concentration of DTT is sufficient to reduce the thiols in low-input samples, however, for simplicity, we chose to continue utilizing 5 mM DTT during the reduction step. Notably, alternative reducing agents such as tris(2-carboxyethyl)phosphine (TCEP) are commonly employed for thiol reduction and show advantages in specific applications, which could be another choice for the reducing step in our workflow.

In addition to the vacuum manifold method, an identical set of samples was enriched using a 96-well filtered deep-well plate with our custom-molded plate seal. Similar results were obtained, showing little impact of DTT on cysteine-containing peptide identification and comparable numbers between the vacuum manifold and well-plate methods (Supplemental Fig. S2, *A* and *B*). Furthermore, we also tested the impact of DTT on total thiol samples with both enrichment methods. In both cases, samples reduced with 5 mM DTT yielded more peptides and proteins than samples reduced with 0.5 or 1 mM DTT (Supplemental Fig. S2, *C* and *D*). Given an average of 10% oxidation (36), total thiol samples are expected to have a 10-fold higher concentration of cysteine-containing peptides than the total oxidation samples, requiring a higher concentration of DTT for complete reduction.

We then explored whether there was an optimal resin amount for enriching low-input samples. To maintain the same resin-to-peptide ratio in our established protocol (30 mg of resin to 200 μg of peptides), we would need to use <1 mg of resin for low-input samples. Given that a small amount of resin is hard for robust handling and reproducibility, we chose to test 2.5, 5, and 10 mg of resin for 1 to 5 μg of peptide input. Such ratios are not expected to be capacity-limiting for enriching cysteine-containing peptides (*e.g*., estimated ∼23,000-fold molar excess of ligand on 10 mg of resin for 2.5 μg input). While all amounts performed similarly, 10 mg of resin produced the most consistent results and yielded slightly more peptides than 5 or 2.5 mg (Figs. 3*A* and Supplemental Table S3). At the protein level, 10 mg also yielded slightly higher number of identifications for 2.5 and 5 μg of peptide input (Fig. 3*B*).

Based on these results, the optimal enrichment conditions were 5 mM DTT in the reduction step (in a 100 μl binding reaction volume) and 10 mg of thiol-affinity resin per sample for 1 to 5 μg of peptide input. It should be noted that other parameters—such as the sample type/complexity, number of washes, binding reaction volume, resin type, and alternative desalting strategies—may also affect enrichment efficiency. During the development of this work, we tested enrichments from two different cell types as well as mouse muscle tissue, in which no adverse issues were encountered. Since the enrichment in this workflow is performed at the peptide level, which is generally more homogeneous than protein-level enrichment, it is expected to work with other complex sample sources.

### FAIMS Increase the Sensitivity of Low-Input Redox Proteomics

During the development of this protocol, we observed singly charged polymer peaks, originating either from the enrichment resin or sample preparation tubes, in enriched samples from low input amounts. Although these polymer peaks were also detectable in standard input samples (*e.g*., 200 μg), their intensities were lower relative to the peptide signal. However, in low-input samples, such species can suppress the ionization of peptides and hinder peptide and protein identification. To mitigate their impact, we used high-field asymmetric waveform ion mobility spectrometry (FAIMS), which can effectively remove singly charged interference ions by applying an asymmetric waveform and a compensation voltage (Fig. 4*A*) (39). FAIMS has been successfully applied to numerous single-cell and low-abundance (PTM) proteomics workflows (40, 41, 42). We first ran technical replicates of the equivalent of 20 μg peptide input using CVs ranging from 40 to 70 V and collected DDA spectra with a 30 min LC gradient. We found that dual CV stepping at 45 and 60 yielded the highest number of unique peptide identifications for both total oxidation and total thiol samples (Supplemental Fig. S2 and Supplemental Table S4). Since the data were collected with a 30 min LC gradient, we noted that the number of unique peptides was lower than in previous experiments that used a 2 h gradient. Applying the optimal CV setting to samples containing 1 to 5 μg of peptides resulted in an average increase of 145.6% in cysteine-containing peptide identifications (Fig. 4*B* and Supplemental Table S5) and 92.8% in protein identifications (Fig. 4*C*). As an example, FAIMS enabled the detection of a cysteine-containing peptide from GAPDH (Glyceraldehyde-3-phosphate dehydrogenase), which was undetectable without FAIMS (Supplemental Fig. S4). Additionally, the intensity of the +1 m/z contaminant peaks was significantly lower in the FAIMS runs compared with non-FAIMS runs (Supplemental Fig. S5). We conclude that FAIMS effectively reduces interfering, singly charged polymer ions, thereby improving peptide and protein identifications in low-input redox proteomics samples. We therefore employed these FAIMS conditions in all subsequent low-input experiments.

### Redox Proteomics of Cells Cultured in 96-Wells

To demonstrate the utility of our workflow, we processed a full 96-well plate of RAW cells using the low-input, high-throughput SP3 method coupled with RAC enrichment (Supplemental Fig. S6*A*). Cells were seeded at densities of 10,000 or 40,000 cells per well and treated with 1 mM diamide for 30 min to induce oxidative stress. Starting from cell lysis, it took approximately 2 h of hands-on time for SP3 to generate peptides. BCA analysis showed the recovery of ∼4 μg and ∼8 μg of peptides after SP3 elution from the 10K and 40K cells, respectively (Supplemental Fig. S6*B*), demonstrating the high recovery of the plate-based lysis method. It took another 2 h of hands-on time for SPE and enrichment for all 96 samples. Twelve peptide samples representing the four experimental groups (two cell densities and two treatment conditions; n = 3 per group) were randomly selected for DDA analysis, which identified a total of 5983 unique cysteine-containing peptides (Supplemental Table S6). However, a substantial proportion of missing values was observed, with only 474 peptides quantified in at least two of three replicates for both diamide-treated and control 40K cell groups.

Because DIA systematically fragments all precursors within defined m/z windows, it reduces stochastic sampling and missing values relative to DDA, thereby improving quantification completeness in low-input proteomics (43). To improve quantification completeness, another set of 12 samples was analyzed by DIA, resulting in the identification of 13,543 cysteine-containing peptides corresponding to 10,682 unique cysteine sites across 4235 proteins (Supplemental Table S6). Identification confidence was further evaluated by examining NEM-modified peptides, which in our workflow are expected to be enriched primarily when they also contain an additional cysteine with a free thiol (*i.e*., originating from an endogenously oxidized site). Using target–decoy results to calculate per-run FDRs, we observed the expected global peptide-level FDR of 1% (as specified in the search) and a modestly higher modification-specific FDR for CysNEM peptide (mean 1.65%). Importantly, our redox readout is based on peptides containing unmodified cysteines after DTT reduction, for which the FDR remained ∼1%. Quantitatively, 3015 unique cysteine sites were quantifiable (present in at least two of three replicates per condition) in the 40K cell samples, and statistical analysis revealed an overall more oxidized redox proteome in diamide-treated cells than in controls (Supplemental Fig. S6*C*). For the 10K-cell samples, 1637 sites were quantifiable with a similar overall right-shift volcano plot shape (Supplemental Fig. S6*D*). While the coverage in DDA for these samples was insufficient, our 40k cell cultures produced robust data. With high-throughput cell culture scales at 40K density for many cell types, we expect that employing the plate-based cell culture of 40K cells, low-input redox sample-processing workflow, and DIA will find broad application in the field.

To evaluate whether the performance was maintained beyond a single 96-well plate run, we next assessed the robustness of the plate-based, low-input SP3–RAC workflow. Specifically, we repeated the same experimental design across three independent batches, each batch consisting of 96 samples on a plate (40K cells per well), half treated with DMSO and the other half with diamide. For each batch, SP3-RAC was performed for the whole plate and six replicates from each of the two treatment groups were randomly selected for DIA runs. Importantly, the three batches of cells were cultured, treated, and processed on different days to test any batch effect. Across the three batches, the numbers of quantified peptides, cysteine-containing peptides, and proteins per sample were comparable within each condition (*e.g*., an average of ∼3600 quantifiable cysteine-containing peptides in DMSO and ∼6500 in diamide; Supplemental Fig. S7*A*), indicating consistent coverage and good reproducibility across batches. In addition, diamide-treated samples yielded higher identification counts than DMSO controls across all three metrics, consistent with diamide-induced oxidative stress increasing cysteine oxidation and thereby improving the detection of cysteine-containing peptides. Next, peptide-level quantitative reproducibility was assessed by calculating coefficients of variation (CVs) across the replicates within each batch and condition, which similar overall CV distributions across batches with an average median CV of 40% (Supplemental Fig. S7*B*).

**Fig. 5:**
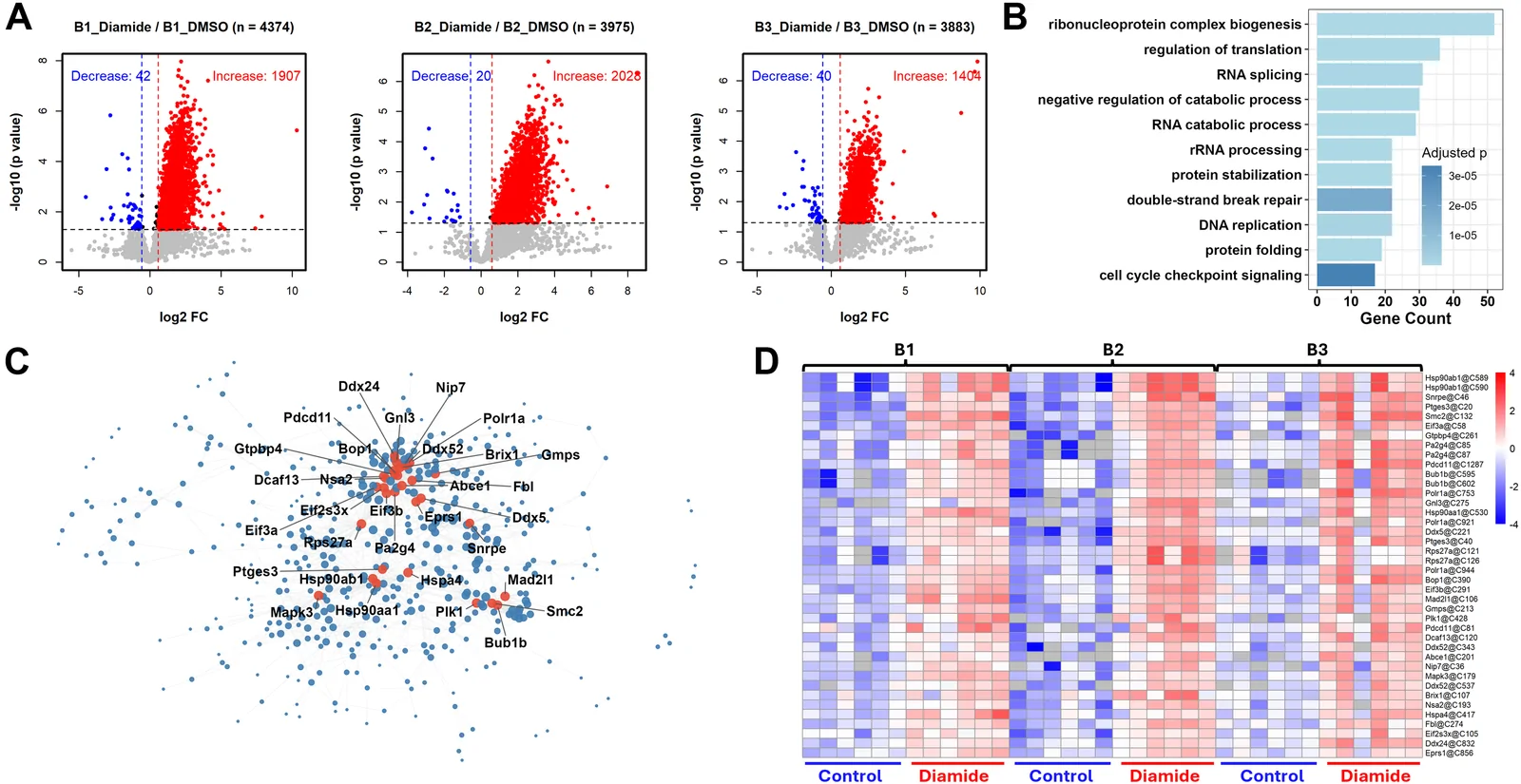
**Impact of diamide on the redox proteome using 40K-cell input and DIA quantification.***A*, Volcano plot showing differential cysteine oxidation between diamide-treated and control cells. For each of the three batches (B1 to B3), six replicates were included for each condition (control and diamide). Changes in oxidation levels of unique cysteine sites between the diamide and control are shown. *Dashed blue* and *red vertical lines* indicate log_2_ fold change cutoffs of −0.26 and 0.26, respectively, and the horizontal *black line* indicates a *p*-value cutoff of 0.01. *B*, enriched biological processes identified from proteins showing significant changes in cysteine oxidation across the three batches. *C*, protein–protein interaction network of significantly oxidized proteins, with hub proteins highlighted in *red*. *D*, heatmap showing oxidation levels of hub proteins at individual cysteine sites. For each site, log_2_-transformed values were scaled by the mean across all samples. *Gray* indicates missing values.

We next evaluated whether the three batches revealed consistent biological effects of diamide on the redox proteome. Differential analysis within each batch yielded a highly consistent response, with the vast majority of significant sites showing increased oxidation upon diamide treatment (Fig. 5*A*). The numbers of significantly increased sites were comparable across batches (1907, 2028, and 1404 in batches 1–3, respectively). UpSet analysis further showed that 1982 of 2944 significant cysteine sites (67.3%) were detected in at least two batches, including 774 sites shared across all three (Supplemental Fig. S8). Moreover, effect sizes for shared sites were positively correlated across batches (mean Pearson r for log2 fold-changes = 0.55; Supplemental Fig. S9), indicating that DIA enables reproducible quantification of diamide-induced cysteine oxidation from low-input samples across independent plates and processing days.

### Redox-Sensitive Cysteines and Proteins to Diamide Treatment

Having established that our plate-based low-input redox proteomics can capture consistent oxidation signatures across three independent batches, we next asked which proteins and pathways were most reproducibly affected by diamide. Proteins with significantly increased thiol oxidation observed in all three batches (n = 777 sites on 590 proteins) were enriched in redox-sensitive biological processes such as translation, DNA replication, and protein folding (Fig. 5*B*). During oxidative stress, free-radicals react indiscriminately with nearby molecules, causing extensive damage that necessitates repair, refolding, and *de novo* synthesis of proteins and RNAs to replace the damaged cellular components (9, 44, 45). Thus, the enrichment of proteins involved in DNA biosynthesis, ribonucleoprotein complex biogenesis, chaperoning and hydrogen peroxide metabolic processes (Fig. 5*B* and Supplemental Table S7) supports the validity of our low-input redox proteomics analysis.

Network analysis of more oxidized proteins further highlighted hub proteins with extensive interactions (Fig. 5*C*, which shows the names of hub proteins; and Supplemental Fig. S10 showing the names of all proteins), whose oxidation under oxidative stress may alter their interactions with client proteins and thereby modulate downstream cellular activities. We next ranked the hub proteins by the degree of fold change under diamide treatment and investigated their roles in oxidative stress response pathways (Fig. 5*D*). The top hit Hsp90ab1 has been established as a critical chaperone protein implicated in client protein stabilization, redox sensing *via* cysteine PTMs under oxidative conditions, apoptosis modulation, and co-chaperoning (46, 47, 48) (*e*.*g*., with Hspa4, also present in our enriched heatmap). Furthermore, client kinases of Hsp90 are also enriched, including Plk1 (49), Mapk3 (50), and Bub1b (51). The next highest fold-change protein is Snrpe, a small ribonucleoprotein which is a core component of the spliceosome as well as a known interactor with Hsp90 (52). The consistent identification of Hsp90 isoforms, Hsa4, and other chaperone hub proteins in response to diamide aligns with prior studies showing chaperones play key roles under oxidative stress (53, 54, 55, 56). The third highest fold-change protein, Ptges3, is a cytosolic prostaglandin synthase and possesses glutathione S-transferase (GST) like activity (57). Glutathione is present in very high concentrations within the cell and is major a redox-active molecule. GSTs serve to conjugate glutathione to xenobiotics and reactive oxygen species (ROS) like hydrogen peroxide to quench overoxidation. Thus, this data suggests a role cysteine oxidation of Ptges3 in detoxification under oxidative conditions. In addition to Snrpe, other top hub proteins are also involved in ribosome, spliceosome recruitment and synthesis (58, 59). During oxidative stress there is significant overhaul in protein expression patterns in response to the oxidative insult (60). Consequently, it is expected that RNA-processing proteins such as these are upregulated under diamide treatment due to the required RNA manipulation for *de novo* protein expression in response to insult. Furthermore, as the ribosome is damaged by oxidative stress, new RNA and protein must be synthesized to build more functional ribosomes.

To contextualize the depth of the redox proteome enabled by the low-input workflow with respect to cellular protein abundance, we assessed coverage of two protein classes that are typically expressed at relatively low levels: transcription factors (TFs) and protein kinases (PKs). Using curated mouse reference lists (1482 TFs; 499 PKs), our RAW-cell dataset covered 95 TFs and 132 PKs (Supplemental Table S8). Notably, many TF and PK cysteine sites displayed increased oxidation upon diamide treatment across all three batches (Supplemental Fig. S11), including TFs involved in stress signaling (*e*.*g*., Rel, Stat1, and Irf3) and kinases implicated in stress and cytoskeletal pathways (*e*.*g*., Map3k2, Rock1/2, and Lyn). To test whether cysteine-peptide enrichment is biased toward highly abundant proteins, we compared cysteine-oxidation signal to protein abundance estimated from non-cysteine-containing peptides. Both rank-based and intensity-based comparisons showed weak association between protein abundance and cysteine-oxidation levels (r = 0.24 and 0.37, respectively; Supplemental Fig. S12), indicating that cysteine-peptide enrichment is not strongly restricted to the most abundant proteins and supports broad proteome coverage rather than a high-abundance bias.

To benchmark our results against prior redox proteomics data, we compared our low-input diamide-treated RAW264.7 dataset with the published site-resolved S-glutathionylation (SSG) study using hundreds of micrograms of protein as the input with a similar diamide treatment in the same cell type (26). The previous study quantified SSG sites by blocking free thiols, selectively reducing glutathionylated cysteines, and capturing nascent thiols on resin followed by on-resin digestion and iTRAQ-based MS analysis. Despite differences in the specific redox types interrogated (total cysteine oxidation in this study *versus* SSG-specific enrichment) and in acquisition/quantification strategies, we observed substantial concordance at the protein level (551 shared proteins), while overlap at the exact cysteine-site level was limited (401 shared sites; Supplemental Fig. S13).

Collectively, these results demonstrate that our high-throughput method generates reliable redox proteomics data from 96-well cell culture samples using only a few μg of protein. Cell culture samples were used for development, but the method should be readily applicable to tissue lysates. While 96 samples were processed here, three to four 96-well plates can be handled in parallel without increasing total time and effort by more than 15%. Future efforts should increase the robustness of the workflow and reduce missing data across samples. Missing values can arise from peptide loss during protein extraction, digestion, or enrichment steps, a challenge that is particularly pronounced in low-input proteomics. The severe missingness observed in DDA datasets reflects the stochastic nature of precursor selection, whereas DIA substantially reduces missingness by systematically fragmenting all ions across runs. Thus, future improvements in sample handling (*e*.*g*., small processing volume) combined with DIA-based and TMT-based acquisition strategies should be evaluated to achieve more robust PTM quantification in low-input studies. With the recent surge of interest in redox signaling and homeostasis for drug development and pathology studies, our approach provides a streamlined and standardized workflow for cell lysis, digestion, and enrichment. This protocol can be used to screen chemical and biological libraries to study their effects on cellular redox status. High-throughput screening is one of the most powerful contemporary biological capabilities, generating large-scale datasets that drive drug mechanistic studies and target discovery. However, redox proteomics, like many other PTM proteomics areas, has been limited by low data availability due to challenging sample isolation, preparation, and enrichment. Our innovative and scalable sample preparation protocol addresses these bottlenecks and provides a foundation for expanding PTM proteomics into high-throughput applications. Future studies will adapt this methodology to other PTMs within our multi-PTM platform.

## Conclusion

The work addresses two major challenges in PTM proteomics. First, scaling down to just a few micrograms of protein reduces the cost and complexity associated with using larger sample inputs. Second, the plate-based sample preparation allows processing of over tenfold more samples weekly than previously possible. This enables the parallel screening of hundreds to thousands of conditions, facilitating the evaluation of molecular libraries for their effects on redox signaling and the development of small-molecule drugs targeting redox PTMs. While further protocol refinement will be needed to adapt the workflow to different sample or PTM types, this study demonstrates the feasibility of reducing input amounts while increasing throughput. Higher sample throughput allows the exploration of far more conditions in parallel and the enrichment of multiple PTM motifs from a single sample source.

## Data availability

All proteomics data generated for this study have been deposited in the MassIVE repository (massive-ftp.ucsd.edu). The dataset can be accessed using the identifier MSV000099923 after entering the username MSV000099923 and password Redox7169.

## Supplemental Data

This article contains supplemental data.

## Conflict of interest

The authors declare that they have no conflicts of interest with the contents of this article.

## Supplemental Information

Supplemental Table S1 Summary of the low input sensitivity test.

Supplemental Table S2 Effect of DTT on redox enrichment.

Supplemental Table S3 Effect of resin amount on redox enrichment.

Supplemental Table S4 Summary of FAIMIS CV optimization.

Supplemental Table S5 Impact of FAIMS on low-input redox proteomics.

Supplemental Table S6 Summary of redox proteomics in diamide-treated RAW cells.

Supplemental Table S7 Summary of three-batch diamide-treated RAW cells experiment.

Supplemental Table S8 Transcription factors and protein kinases in the RAW cell dataset.
