# Supplementary material for: A Scalable Low-Input Redox Proteomics Workflow Enables High-Throughput Analysis of Cysteine Oxidation in 96-Well Cell Cultures
Source: Mol Cell Proteomics. 2026 Aug 17;25(9):101641. doi: 10.1016/j.mcpro.2026.101641 (PMC13590100; doi:10.1016/j.mcpro.2026.101641)
Supplement: Supplementary file 1 [file mmc9.docx]

**A Scalable Low-Input Redox Proteomics Workflow Enables High-Throughput Analysis of Cysteine Oxidation in 96-Well Cell Cultures**

Adam M. Kabza ^a^, Rosey Chu ^a^, Jesse Trejo ^a^, Xiaolu Li ^a^, Matthew J. Gaffrey ^a^, David Hall ^a^, Wei-Jun Qian ^a^, and Tong Zhang ^a*^

a. Biological Sciences Division, Pacific Northwest National Laboratory, Richland, WA, USA

* Corresponding Author

Dr. Tong Zhang

Biological Sciences Division

Pacific Northwest National Laboratory

Richland, WA 99352

**Tel:** (509)371-7780

**Email:** tong.zhang@pnnl.gov

**Supplemental Figure 1. Custom silicone matt press-molded.** (**A**) Top view of an AccroPrep well-plate adjacent to the sealing silicone mat. (**B**) Forward view showing how the plate-holes and mat-plugs align. (**C**) Side view of the plate with the sealing mat mounted sitting on a benchtop. (**D**) Top-angle view of how the silicone mat ‘sticks’ to the plate once pressed/rolled on with gentle pressure.

**A**

**B**

**C**

**D**

**Supplemental Figure 2.** The effects of DTT concentration on enrichment of cysteine-containing peptide (A & C) and protein (B & D) identifications. 5 µg of peptide input was used in each sample. We additionally compare the difference between using individual tubes in the manual protocol (red bars) and a well plate (blue bars). A and B are for total oxidation (TO) samples, and C and D are for total thiol (TT) samples.

**Supplemental Figure 3. FAIMS conditions optimization.** Technical replicates were analyzed with varying FAIMS compensatory voltages (CVs). A) Total thiol samples. B) Total oxidation samples. * denote statistical significance between each of the FAIMS groups to the no FAIMS control ( p < 0.05). Raw data and statistical results can be found in **Supplemental Table S4**.

**Supplemental Figure 4**. **FAIMS increase sensitivity in the detection of enriched peptides.** The Extracted Ion Chromatogram (XIC) was obtained by filtering m/z of 1026.172 mz from the ThermoRAW mass spectrometry files, CV -40V. The precursor corresponds to the peptide sequence VVSTDFNGNPHSSIFDAKAGIALNDNFVK of Gapdh.

**FAIMS OFF – significant +1 m/z contaminants**

**FAIMS ON – elimination of majority +1 m/z contaminants**

**A**

**B**

**Supplemental Figure 5.** Use of FAIMS effectively filters out +1 m/z contaminant ions, improving peptide ID count. Without FAIMS, the +1 m/z contaminant suppresses the peptide signal intensity. +1 charged species are marked with red flags on the spectrum. These spectra are from 5 ug peptide input total oxidation samples. The top spectra and bottom spectra are two samples from the same triplicate group, one run without FAIMS (**A**) and one run with FAIMS (**B**). We have selected XICs with identical retention times to demonstrate how FAIMS filters out +1 charged species and true peptide signal is recovered.

**Supplemental Figure 6. Performance evaluation of full-plate cell culture, SP3 sample processing, and redox proteomics.** (**A**) Cell culture plate seeding and treatment layout of RAW 264.7 macrophage cells. Samples from this plate were taken at random (three for each group) and used for DDA and DIA experiments. (**B**) Recovery of proteins and peptides from 10K and 40K cells. BCA quantification of protein (at lysis) and peptide (after digestion and elution from SP3) for low input samples demonstrating that enough material is retained through SP3 handling to continue on to effective redox enrichment on the thiol-affinity resin. (**C-D**) Volcano plots showing differential cysteine oxidation between diamide-treated and control in 40K (**C**) and 10K cells (**D**). Three replicates were performed for each condition. Dashed blue and red vertical lines indicate log₂ fold change cutoffs of −0.26 and 0.26, respectively, and the horizontal black line indicates a p-value cutoff of 0.01. The total number of quantifiable sites (at least two valid values in each of the comparing groups) was indicated in the plot title.

**Supplemental Figure 7. Identification depth and reproducibility across three batches using the plate-based low-input redox proteomics approach.** (**A**) Barplot showing the quantified identifications for peptides (light blue), cysteine-containing peptides (orange), and proteins (purple) in each batch (B1–B3) under DMSO control or diamide treatment. Bars show the mean across six replicates (R1–R6) and error bars indicate ± SD; individual replicate values are shown as black dots. Blue numbers above bars denote the mean counts. (**B**) Distributions of peptide-level coefficients of variation (CV, %) within each batch and condition group. Blue labels indicate the median CV for each group.

**Supplemental Figure 8**. **Overlap of significantly oxidized cysteine sites across three batches.** The results of differential analysis comparing diamide-treated and DMSO control samples in each batch (B1–B3) were summarized. Horizontal bars on the left indicate the total number of significant sites in each batch (set size). Vertical bars indicate the number of sites shared among the batch combinations.

**Supplemental Figure 9. Cross-batch concordance of differential expression effect sizes.** Pairwise scatter plots show log2 fold-changes (log2FC) between batches. Each point represents a cysteine oxidation site (common significant sites among the three batches, filtered by p < 0.01 and absolute log2FC above 0.5). The dashed line indicates y = x. The solid red line shows the least-squares linear fit. Pearson correlation coefficients (r) are shown in each panel.

**Supplemental Figure 10**. Protein–protein interaction network of significantly oxidized proteins. All 590 proteins from the 774 cysteine sites showing significant increase in oxidation across three batches (**Fig. S8**) were used for the network construction and labeled.

**Supplemental Figure 11**. **Diamide-induced oxidation of cysteine sites in low-abundance regulatory proteins.** Heatmaps of significant oxidized cysteine sites identified in all three batches in RAW 264.7 cells for transcription factors (TFs; **A**) and protein kinases (PKs; **B**) were shown. For both panels, samples are grouped by batch (B1–B3) with control (DMSO) and diamide conditions shown for each batch (n = 6 wells per condition per batch). Values are row-scaled (z-score) log2 intensities per cysteine site, with red indicating higher and blue indicating lower relative oxidation signal across samples. Site labels are shown as Gene@Cys position.

**Figure S12. Relationship between protein abundance and cysteine-oxidation levels.** Scatterplots comparing protein abundance estimated from non-cysteine–containing peptides to cysteine-oxidation. Left: comparison of abundance ranks (rank 1 = highest). Right: comparison of mean log2 intensities. The abundance values were obtained by summing peptide intensities to the same protein. Pearson correlation coefficients are shown in blue (r = 0.24 for ranks; r = 0.37 for mean log2 intensities).

**Supplemental Figure 13**. **Overlap of redox proteome coverage between this study and published data.** Venn diagrams showing the overlap of quantified cysteine oxidation sites (**A**) and (**B**) proteins between this study and the published dataset from Su et al. Both studies performed diamide perturbation in RAW267.4 cells.
